# Surveillance of Infectious Diseases by the Sentinel Laboratory Network in Belgium: 30 Years of Continuous Improvement

**DOI:** 10.1371/journal.pone.0160429

**Published:** 2016-08-29

**Authors:** Gaëtan Muyldermans, Geneviève Ducoffre, Mathias Leroy, Yves Dupont, Sophie Quolin, participating sentinel laboratories

**Affiliations:** WIV-ISP, OD Public Health and Surveillance, Unit ‘Epidemiology of infectious diseases’, Brussels, Belgium; Centers for Disease Control, TAIWAN

## Abstract

In 1983 the sentinel laboratory network was established because of the need to describe the epidemiological evolution of infectious diseases. During the study period of 30 years (1983–2013), microbiology laboratories reported on weekly basis the laboratory diagnosed cases for a selection of infectious diseases. This resulted in a large longitudinal laboratory based database allowing to provide trends over time and distribution by person and place. During this period, adaptations to data collection were made due to changes in diagnostic methods and public health priorities, introduction and application of digital revolution, and multiple reorganizations of the laboratories. Since the surveillance network is dynamic, it necessitates a continuous evaluation to ensure that, over time, it continues to be representative of the general epidemiological trends in the country. Secondly the aim is to examine the robustness and stability of this surveillance system. Here we demonstrated that the flexibility of the data collection methodology by the sentinel laboratory network is unique and that adaptations do not affect the capacity of the system to follow trends. Therefore, the surveillance by this network is representative of the current epidemiological situation in Belgium. To our knowledge, no such surveillance network with such a long-term follow-up and demonstrated stability for multiple infectious diseases in the general population was earlier described. Furthermore, expected trends due to the implementation of vaccination or other events were accurately detected. The collected data obtained from this network allows interesting comparisons with other national and international information sources.

## Introduction

In Belgium, the sentinel laboratory network was established in 1983 in order to obtain information on the epidemiology of infectious diseases [1]. The laboratories participating to this network are further called the sentinel laboratories.

This sentinel laboratory network is coordinated by the Scientific Institute of Public Health (WIV-ISP) organizing the data collection and data storage, and facilitating the data processing and dissemination of this information.

The main objective of this network is to monitor the evolution of different infectious diseases over time, both within a calendar year and over several years. The collected information allows as well to fulfil national and international (i.e. ECDC, WHO) requests.

Next to this sentinel laboratory network, other surveillance networks for human infectious diseases, complementing each other, are available in Belgium, i.e. the notification of infectious diseases organised by the Flemish Community [2], Brussels Capital and the French-speaking Community [3], the network of paediatrics collecting mainly data on vaccine preventable infectious diseases in children since 2002 [4], the network for surveillance of sexually transmitted diseases since 2000 [5], the network of national reference laboratories and the national reference centers collecting public health microbiology data since 2011 (see also materials and methods) [6] and the registration network of general practitioners since 1979 [7]. Each surveillance system has its strengths and weaknesses.

Although the mandatory notification system and the sentinel laboratory network are both fed by microbiology laboratories, additional clinical information is provided to the mandatory notification system by the treating physicians which renders this system exhaustive. On the other hand, the sentinel laboratory network is based on a fraction of the microbiology laboratories raising concerns about the representativeness nationwide and regional [8].

By monitoring 12 pathogens, it was previously demonstrated [9,10] that the coverage of the sentinel network was stable over time and close to, or greater than 50%. Test coverage was in this study calculated by the ratio of reimbursed tests performed by participating laboratories to the total number of tests performed. These results indicate that the network is sensitive and representative for the surveillance of the selected pathogens. Furthermore, these results hold for the 3 regions of Belgium but at the provincial level, a lower test coverage was shown for some pathogens [10]. Moreover, the information provided by the sentinel network is usually considered to be timelier and more complete due to a better compliance of voluntary reporting laboratories [8].

This paper describes the historic changes and developments of the Belgian sentinel laboratory network such as the flexibility of adaptation of the network towards pathogen changes, diagnostic method changes, data transfer methodology and multiple reorganization of the participating sentinel laboratories. The lack of impact of these changes on the robustness of the longitudinal surveillance of this network was investigated by a detailed description of the trend changes for a series of infectious diseases.

## Materials and Methods

### Organisation of the network

The sentinel laboratory network was implemented from 1983 onwards as described previously by Walckiers et al. (1991) [1]. Briefly, microbiology laboratories transferred on a weekly basis their laboratory diagnosed cases to the WIV-ISP on paper form by regular mail. The information for a limited number of variables was collected for a selection of infectious diseases. The encoded variables included the diagnosed infectious disease, some patients demographic data allowing the identification of duplicates i.e. date of birth (or previously age), gender and postal code. In addition the specimen and its sample identification number, the diagnostic method and the date of diagnosis are recorded as well. If applicable, the registration of the country of infection is foreseen. For confidence reasons, all data transfers are kept anonymous towards the patient.

All data were collected in a central database and analysed for a quarterly and annual report. The participation by the sentinel laboratories was and is still today voluntary and without remuneration.

If requested by a dedicated reference laboratory, the sentinel laboratories are encouraged to send strains to the reference laboratories/centers for further characterisation such as geno- and/or phenotyping or antimicrobial follow-up.

The project on sentinel laboratory network is scientifically supervised by a steering committee composed by representatives of the sentinel laboratories, reference laboratories/centers and authorities from the Flemish Community, Brussels Capital and the French-speaking Community having infection control and prevention into their competencies.

The list of infectious diseases is yearly reviewed by this steering committee selecting pathogens based on the current need of public health.

In 2013, the trend analysis included 35 pathogens and covered respiratory infections, gastrointestinal infections, sexually transmitted infections, imported infections as well as zoonosis and vaccine preventable diseases [11]. The list of pathogens is chosen such that it does not overburden the administrative work for the sentinel laboratories.

### Study period

The impact of historical adaptations during the study period of 30 years, starting from the implementation of the project (1983) until 2013, were recorded and described.

### Historical adaptations to improve the functioning of the network

During the study period, the network underwent several adaptations according to the evolution at the level of microbial diagnosis and due to the digital revolution. A summary of all adaptations is briefly described underneath.

Since the implementation of the network, the **diagnostic** methods were yearly revised. As of 1983 all data covered culture positive cases for the 26 infectious diseases. Meanwhile cases diagnosed by serological methods were included gradually since 1987, those by molecular diagnosis (PCR) gradually since 2004 and currently the molecular diagnosis is included for 31 out of the 36 infectious diseases.

The weekly **transfer** of data from the laboratories to the WIV-ISP was initially on paper format. Since the beginning of 2000, some of the laboratories were able to extract the needed information directly from their databases. These cases were recorded in batch to the database. During the same period, other laboratories reported their data via a web application, developed and made available by the WIV-ISP. An online submission tutorial was provided to the participating laboratories extracting their data from their laboratory information management system (LIMS) or those reporting by the webtool. As further described in the results section, the fractions of data obtained by the different data transfer methods by the participating sentinel laboratories changed during the last few years. All historical datasets obtained by the different data transfer methods were collected on a SQL server at the public health institute WIV-ISP for further data cleaning.

Since the microbiology laboratory test results populating the database throughout all these years are neither uniformly coded nor documented in a standardized manner, some **quality assurance measurements** were taken. The application of a systematic approach was introduced since 2009 by the definition of harmonized variables and their formats including well defined own coded values [11]. This was felt necessary for an ongoing oversight and a management of the database to remain valid and useful. All procedures were described in standard operating procedures (SOP) and are available in the internal document management system (not shared in public but available on request).

A number of **quality checks** were gradually implemented to improve the completeness and correctness of the reported cases in the database.

Firstly, the completeness of the recorded cases reported by paper format was double checked by a second person.

Secondly, a monthly feedback was sent since 2012 to all sentinel laboratories to provide an overview of all transferred data since the beginning of the current year. This feedback allows the participating laboratories to determine the completeness of their data in the database and moreover to compare the rate of diagnosed infectious diseases of their laboratories with those from all participating laboratories. This feedback system allows to receive the missing data from the participating laboratories and improving thereby the completeness of the data.

Lastly, quality checks toward variable completeness, consistency, content, alignment with specifications (case definitions), expected trends (seasonal and yearly) are performed on weekly basis for a few cases, selected by cherry picking from all received data.

Recently **case definitions** for each of the infectious diseases were defined [11] based on available international information [12,13] to harmonise the inclusion of cases and to ensure their fulfilment of the case criteria.

The availability of reference laboratories collaborating with this network on a voluntary basis was since 2011 replaced partially by a network of **National Reference Centers** (NRC’s) [6]. These NRC’s were selected based on defined criteria, are reimbursed for their activities and need to fulfil to the proper quality assurance level (ISO15189). The tasks of these laboratories and NRC’s are to diagnose or to confirm rare diseases or diseases difficult to diagnose, to perform some further typing of strains, to determine the resistance towards antimicrobials or their resistance mechanisms and finally provide these epidemiological and microbial data for reporting at national and international levels.

### Data cleaning

The definition of the variables and their formats allowed assembling all historical data into one database on which longitudinal trend analysis can be performed.

Since 2012, a data cleaning program was implemented with SAS software (SAS Institute Inc.^®^, Cary, NC, USA). The program mainly executes the following steps: append all historical tables, harmonize variable names and their format, compute new variables (i.e. age, agegroup), and remove duplicates on basis of sample identification or patient demographic data i.e. date of birth (or previously age), gender and postal code.

The encoded variables included the diagnosed infectious disease, some patient’s demographic data and specimen and analysis method information.

This clean table is optimised by statistical programs and is automatically updated twice a day.

### Data analysis

A web platform called “Epistat” (https://epistat.wiv-isp.be/) was built since 2012 for secured feedback and real time analysis of the clean database. This platform allows the construction of different types of graphs for epidemiological monitoring and for further investigations according to the needs of the sentinel laboratories and other stakeholders: time distributions, geographical maps, age pyramids, pie charts and histograms. Created graphs may be used in surveillance reports and scientific publications by both the WIV-ISP and the sentinel laboratories.

The tool is composed of three different parts: a webform, written in HTML5 and Javascript; a SAS program (SAS Institute Inc. ^®^, Cary, NC, USA) generating the different options appearing in the form; and a SAS stored process receiving the selected options in the form and creating the final output in the browser window. Since the software runs on the web, the application is accessible only with an internet connection and a browser. Access to this application is restricted to the participating laboratories, the WIV-ISP staff members and the officers from the health authorities with logins and passwords.

## Results

### Laboratory participation rate

**Fig 1:**
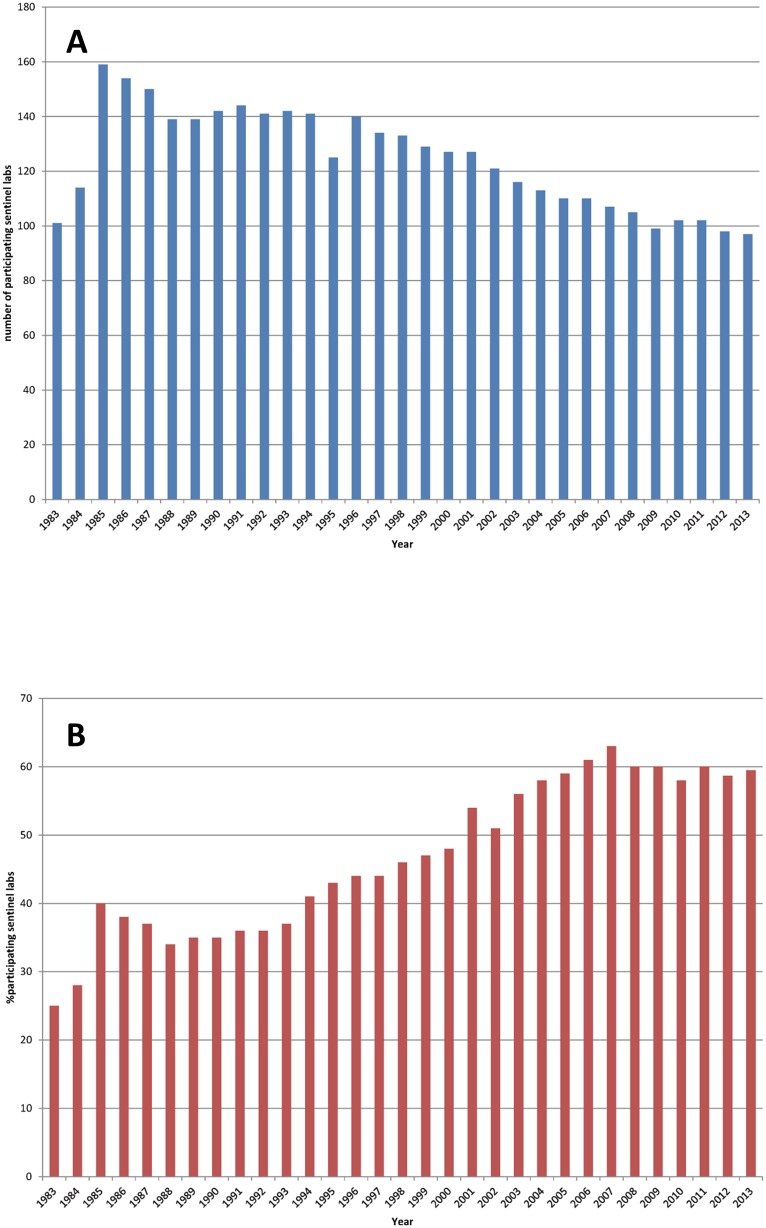
Number of sentinel laboratories participating to the sentinel laboratory network (A) and percentage of microbiology laboratories participating to the network as compared to the total number of registered microbiology laboratories (B).

During the study period, the number of participating laboratories has decreased over time (Fig 1A). After a first introduction period of the network, 159 laboratories participated to the surveillance in 1985 and currently we observed 97 sentinel laboratories in 2013. However due to structural reorganizations and fusions of some clinical laboratories, the ratio of participating sentinel laboratories from all microbiology laboratories has increased from 40% in 1985 to 58% in 2004 and remained constant since then (Fig 1B). In 2013, the fraction of participating laboratories on the total number of registered microbiology laboratories in Belgium was 59% (97 participating laboratories out of 163 microbiology laboratories). We could not determine whether the academic and non-academic hospital related laboratories, and non-hospital related laboratories participating to the network are proportionally representative from the laboratories performing microbiological diagnoses in Belgium.

However, the coverage, a measure of the proportion of target population included in the surveillance system was previously studied [9, 10] demonstrating that the participating laboratories were performing more than 50% of all tests, and that the coverage was constant in Belgium between 1999 and 2002 [9] and between 2007 and 2012 [10].

In 2013, the distribution of sentinel laboratories by region was 54% in the Flemish Community, 34% in the French-speaking Community and 12% in Brussels Capital. This distribution is comparable to that of all the registered microbiology laboratories in Belgium (data not shown) demonstrating its regional representativity. Also the distribution of the Belgian population (n = 11.099.554 in 2013) is similar: 57% in the Flemish Community, 32% in the French-speaking Community and 10% in Brussels Capital.

### Evolution in Number of Pathogens and Data Transfers

The number of infectious diseases for which data were collected, increased from 26 in 1983 to the currently 35.

**Fig 2:**
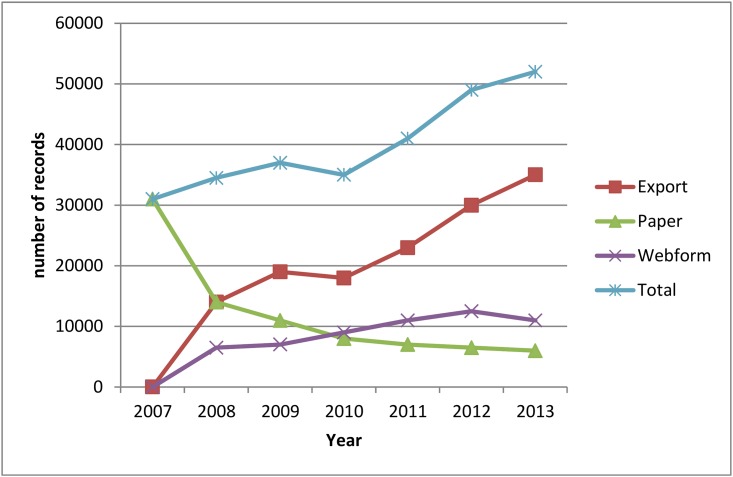
Number of reported cases before removal of the duplicates from 2007 when the digital data transfer became available until 2013. The total number of reported cases (Total) is transferred by sending the information on paper format by regular mail (Paper), by importing the cases via a web application developed by the WIV-ISP (WebForm), or by sending an extraction of the cases from the laboratory information management system (Export).

A gradual increase of the number of reported cases was observed during the study period of 30 years (data not shown). Especially during the last decade when the digital data transfer became available, a tremendous increase of the number of reported cases was observed (Fig 2). The availability of a digital data transfer system reduced the workload substantially and improved the speed of data transmission. However, this necessitates the availability of an automatically process to clean the data especially removing the duplicates. As demonstrated further, the implementation of digital data transfer improved the completeness of the data without impact on the number of cases after removal of duplicates.

In 2013 only 12 laboratories (12%) used the paper form to report their data.

### Long standing follow-up of infectious diseases

**Fig 3:**
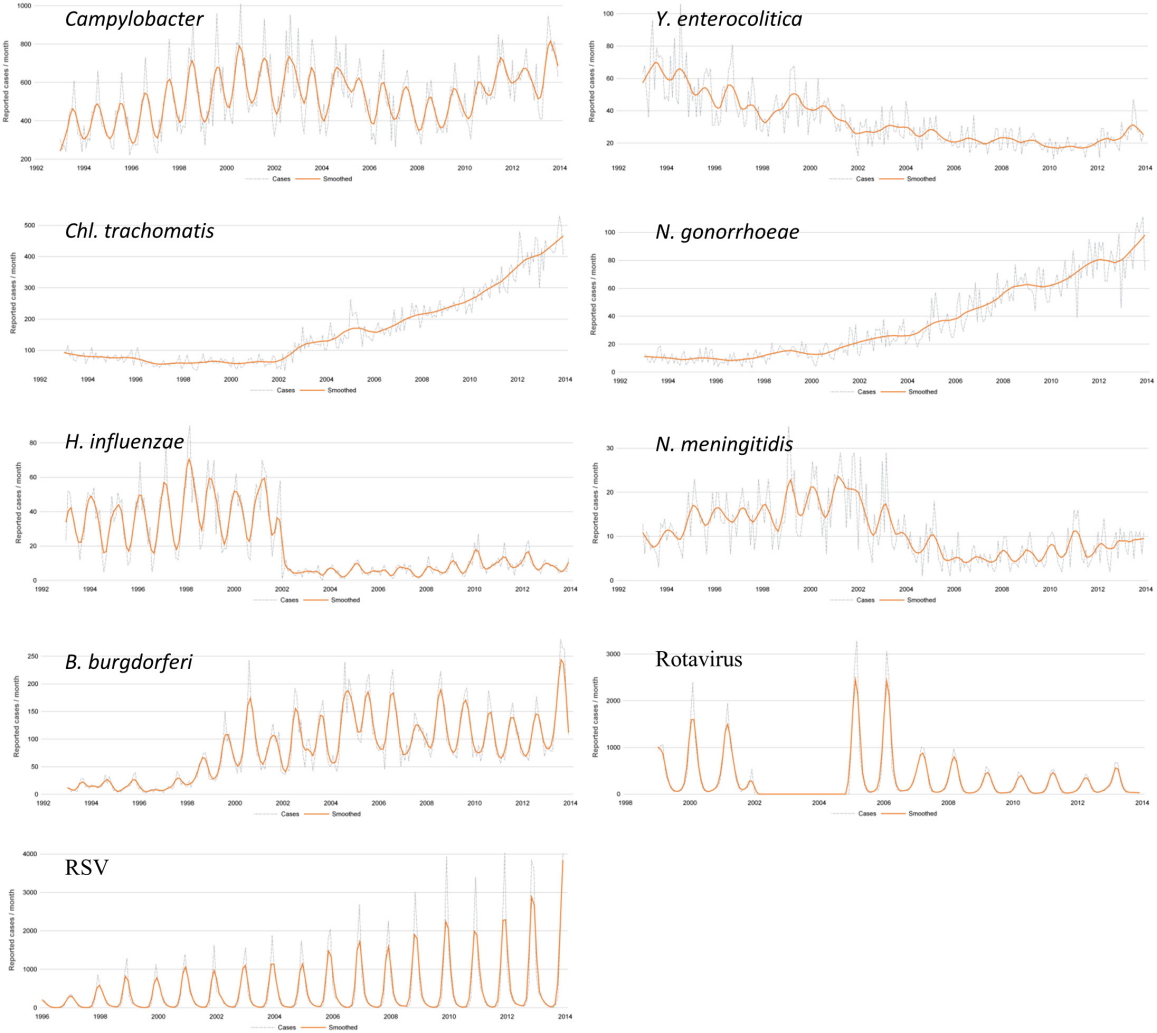
Trend analyses for some representative infectious diseases as measured by the sentinel laboratory network from 1993 (if available) until 2013. The dashed line represents the monthly number of cases while the orange line represents a smoothed curve obtained by the Loess statistical method (SAS Institute Inc. ^®^, Cary, NC, USA), a weighted scatterplot through these data points. *Campylobacter*, *Y*. *enterocolitica*, *Chlamydia trachomatis*, *Neisseria gonorrhoeae*, *Haemophilus influenzae*, *Neisseria meningitidis*, *Borrelia burgdorferi*, Rotavirus, and RSV.

For 10 infectious diseases (*Campylobacter*, *Chlamydia trachomatis*, *Entamoeba histolitica*, *Haemophilus influenzae*, *Legionella pneumophila*, *Neisseria gonorrhoeae*, *N*. *meningitidis*, *Plasmodium*, *Streptococcus pneumoniae*, and *Yersinia enterocolitica)*, the surveillance covered the entire study period of 30 years. Fig 3 demonstrates the yearly trend analysis for some of these including the seasonal activities. Although for *Yersinia enterocolitica* a continuous decrease was observed since 1983, the *Campylobacter* surveillance demonstrated a continuous number of reported cases. Within this waving incidence over the years (slight increase till 2000 followed by a decrease and relapse again from 2010 onwards), we observed a seasonal decrease in summer 1999. During the same period, the contamination of feedstock with polychlorinated biphenyls was demonstrated leading to the destruction of massive amounts of animal food products, mainly eggs and chicken [14]. This so called dioxin affair led to the reduced consumption of these animal food products and thereby a significant decline (40%) in the number of infections [15].

For the follow-up of the sexual transmitted infections (STI), a continuous increase is observed as demonstrated in Fig 3 for *C*. *trachomatis* and *N*. *gonorrhoeae* without notification of a similar increase of reimbursed tests [10]. For *C*. *trachomatis* (in 2001: N = 775, in 2013: N = 5232) the number of cases in the age range of young women from 15 to 29 years explains largely the increase, while for *N*. *gonorrheae* (in 2001: N = 230, in 2013: N = 1063) the increase is situated in particular in men from 20 to 34 years and for *T*. *pallidum* (in 2002: N = 114, in 2013: N = 1293), the increase is situated in particular in men from 35 to 49 years old. These increases are mainly observed in the Flemish Community (district of Antwerp) and in Brussels Capital (data not shown). These evolutions are also observed in other European countries [16]. It is also necessary to remain vigilant watchful as for the evolution of the used techniques of diagnosis to explain our observations. For example, the development since 2002 of more sensitive techniques of diagnosis could partially explain the increased diagnosis of sexual transmitted infections. However, the implementation of a clinical STI network confirmed the increase of STI [17].

For *Haemophilus influenzae* and *Neisseria meningitidis*, a sudden decrease was observed from 2002 onwards. This could be brought in relation with the introduction and/or the reimbursement policy of the vaccines against these infectious diseases. For *Neisseria meningitidis*, a drop from 280 cases in 2001 to 108 cases in 2013 was observed. The reference laboratory/center for *Neisseria meningitidis* receiving the strains for further subtyping, demonstrated the decrease in incidence of serogroup C from 49.4% in 2001 to 10.4% in 2013 [18] showing that the *N*. *meningitidis* serogroup C was brought under control.

For 11 other pathogens (*Borrelia burgdorferi*, *Bordetella*, *Chlamydia psittaci*, *Cryptosporidium*, *Giardia*, Hantavirus, Hepatitis A virus, *Influenza*, *Mycoplasma pneumoniae*, *Streptococcus pyogenes*, and *VTEC)*, a surveillance is available spanning a period of more than 20 years. The trend analysis and other surveillance systems are described in details in the annual reports and are available on the website of the network (https://epidemio.wiv-isp.be).

The serological confirmation of *Borrelia burgdorferi*, to diagnose Lyme borreliose, is also included in the surveillance program from the beginning of the years '90. Between 1998 and 2012 the incidence raised reaching yearly between 1000–1500 cases. Within the period 2007–2012, respectively 120.000 to 280.000 tests were performed per year [10]. Since then, due to multiple discussion forums concerning the so called ‘chronic Lyme disease’ or post-treatment Lyme disease syndrome and an increased interest by the media of the Lyme disease in Belgium, the reported cases increased to 2090 in 2013. Also an increased test frequency was demonstrated during this period [10].

For Rotavirus, cases were recorded from 1999 onwards but due to the workload to transfer the huge number of records, it was interrupted a few years later (2002–2004). It was again initiated in 2005 to obtain a background measurement of the number of diagnosed cases before the introduction of 2 vaccines (Rotarix^®^ [GlaxoSmithKline Biologicals Rixensart, Belgium] in June 2006 and RotaTeq^®^ [Merck&Co., Inc.Whitehouse Station, New Jersey] in June 2007) [19]. The number of diagnosed Rotavirus cases decreased from 9414 cases in 2005 to 2359 cases in 2013, demonstrating the possibility of the network to follow-up the effect of the vaccine policy.

For RSV a continuous seasonal increase of cases was reported, starting every year from week 40 i.e. at the beginning of October. The maximum number of cases per week is generally situated at mid-December. The majority of the cases are diagnosed on young children of less than 5 years. In 2013, 8294 cases were diagnosed by the network.

### Representativity of the data

Although the geographical representativeness was previously investigated [9,10] we further investigated whether all sentinel laboratories participated equally well for each pathogen. Table 1 demonstrates the number of participating laboratories per pathogen in decreasing order. *Campylobacter* was reported at median level over the 5 last years by 91 laboratories. Further, Rotavirus and RSV, 2 pathogens introduced some years after the implementation of the network were reported by respectively 82 and 74 participants.

**Table 1:** Overview of the number of sentinel laboratories reporting a particular infectious disease. The indicated number of reporting laboratories is calculated from the median number of sentinel laboratories reporting cases during the last 5 years of the study period.

Pathogen	Reporting laboratories (last 5 y)	
	Median	Range
***Campylobacter***	91	85–94
**Rotavirus**	82	79–84
**RSV**	74	70–78
***Giardia***	73	69–75
***S*. *pneumoniae***	74	67–77
***Y*. *enterocolitica***	66	58–70
***N*. *gonorrhoeae***	64	61–67
***S*. *pyogenes***	53	51–59
***M*. *pneumoniae***	53	48–54
***C*. *trachomatis***	53	51–55

Considering the respiratory infectious diseases RSV, *M*. *pneumoniae*, Adenovirus, and Parainfluenza virus, the number of reporting laboratories varied respectively with 74, 53, 41 and 17 (data not shown). This implicates that the cross pathogen comparison for symptomatic diseases is hampered by the reporting participation of sentinel laboratories.

## Discussion

There is an increasing need, both at national and international levels, to obtain epidemiological information on many infectious diseases. We demonstrated here that the sentinel laboratory network is an important tool to provide the necessary data and thereby to accomplish these tasks. It provides a robust surveillance for multiple infectious diseases in Belgium and this despite some adaptations throughout these 30 years of the surveillance period. The most important adaptations are: changes in diagnostic methodology by the advent of the PCR technology and the implementation of this sensitive technique to replace mainly the culture, structural reorganisations and fusions between clinical laboratories and the digital revolution, all described in materials and methods. It has been implemented in Belgium since 1983 and was since then one of the most important surveillance network in infectious diseases with a good coverage demonstrating a good sensitivity and geographical representativeness [9, 10]. The collection of a limited number of variables encourages approximately 58% of the available laboratories to voluntarily participate to this network. Whether this high participation rate of laboratories reflects a similar coverage of the Belgian population was not assessed in this study but was previously investigated [10].

During these 30 years of study, the surveillance system demonstrated also a great flexibility by having the capacity to monitor pathogens for the entire period (n = 10) while others (n = 11) were added or removed depending of their public health needs. The longitudinal surveillance of these pathogens was exemplified in the results section for some gastrointestinal infectious diseases (*Y*. *enterocolitica*, *Campylobacter* and Rotavirus), sexually transmitted diseases (*Chlamydia trachomatis* and *N*. *gonorrhoeae*), vaccine preventable diseases (*N*. *meningitidis*, *H*. *influenzae* and Rotavirus) and the respiratory diseases (RSV). For all of these pathogens we could demonstrate a surveillance which is in line with current international findings [20].

For the vaccine preventable diseases, we could determine the impact of the introduction and reimbursement **of the vaccine** policy (*H*. *influenzae*, *N*. *meningitidis* and Rotavirus). For Rotavirus, it was previously demonstrated using the sentinel laboratory records that the infectious season was delayed compared to pre-vaccination seasons [19].

We could demonstrate the effect of some environmental factors such as the dioxin affair when during the summer period of 1999 the contamination of the feedstock resulted in the destruction of massive amounts of animal food products [14]. Also during the same period less animal food products such as eggs and poultry were consumed resulting in a decreased incidence of *Campylobacter* infections [15].

On the other hand the yearly increase in intensity of the seasonal peaks for RSV suggests for an increased incidence during the winter period. No indications of shift of the seasonal peaks neither a broadening of the curves were determined.

The huge increase in incidence of sexually transmitted infections (STI) during the last decade is probably a reflection of the waning of prevention campaign. Why the incidence of the different STI is different according to the gender and age group could not be unravelled by the sentinel network due to the lack of clinical information. This observation is nevertheless confirmed by data from other national networks [17].

We demonstrated as well that this network has the characteristic to be dynamic. This feature is supported by the fact that it is possible to add a germ to the content of the recording at any time in the course of the year. Therefore it requires a regular follow-up which is assured by the scientific advice given by the yearly steering committee of the network bringing together the sentinel laboratories, the reference centers, the epidemiologists and the sponsors of the network. The addition of a pathogen as proposed by the steering committee can very easily be added to the list and after informing the participating laboratories, data can be collected without further investments in partners, infrastructure, or methodology of working.

It is also necessary to remain vigilant in continuing the surveillances even if a low incidence is monitored. For instance the low burden of sexually transmitted infections during the 90’s [21] was tempting to remove them from the list. Those years the decrease in incidence was a profit from the massive prevention campaigns for HIV/AIDS. The continuous monitoring of three main sexually transmitted infectious diseases demonstrates that monitoring even at low burden remains important for the long term surveillance.

The roles of the reference centers are to confirm the diagnosis of the received samples and to supply these with other invaluable microbial information, such as the type of circulating strains at the human and/or food level or their sensibility towards antimicrobials. By collecting and providing these microbial informations, the surveillance of particular pathogens is further accomplished. As an example, the reference centers have shown the effect of the vaccination on the decrease of *N*. *meningitidis*, in particular the serogroup C present in the vaccine [18].

A high participation rate (more than 50%) was measured for 10 pathogens. However we found different levels of participation for different infectious diseases belonging to the same symptomatic disease. This is true for the respiratory infectious diseases, but is also true for the gastrointestinal diseases and the sexually transmitted diseases (data not shown). This difference in participation rate between pathogens hampers cross pathogen comparison for infectious diseases belonging to the same symptomatic disease. By the implementation in the near future of a new electronic data transfer system based on harmonized national coding standards and a common data transfer route for all laboratories [22], the partial registration by some laboratories will be overcome.

The advantage of the limited number of variables asked to the participating laboratories makes it easier to stimulate them to participate. It allowed the monitoring of the epidemiology in time and place. The drawback of this limited information is that no clinical information is requested and thus no clinical surveillance can be provided.

In conclusion, the data supplied by this network represent a unique source of information from the point of view of the public health. It allowed us to accurately detect and describe trends, contribute to the estimation of the burden of disease and unravel the effect of the implementation of a vaccine policy or other events. These observations are currently presented in pathogen specific reports and allow furthermore interesting comparisons with other national and international information sources.
